# Complete Genome Sequence of a Novel Circovirus from Zebra Finch

**DOI:** 10.1128/genomeA.00560-15

**Published:** 2015-05-28

**Authors:** Monika Rinder, Anna Schmitz, Andrea Peschel, Rüdiger Korbel

**Affiliations:** Center for Clinical Veterinary Medicine, Clinic for Birds, Reptiles, Amphibians and Ornamental Fish, LMU Munich, Oberschleissheim, Germany

## Abstract

A novel circovirus was identified in zebra finches (*Taeniopygia guttata*). The genome of the circovirus strain, designated 8454V25-1, comprised 1,982 nucleotides with two major open reading frames encoding a replication-associated protein and a viral capsid protein.

## GENOME ANNOUNCEMENT

Circoviruses are small nonenveloped viruses with a circular single-stranded DNA genome of about 2 kb in size, organized in an ambisense direction. Two major open reading frames (ORFs) were identified with a replication-associated (Rep) protein encoded on the viral strand (V1) and a capsid protein (Cap) encoded on the complementary strand (C1) (1). The genus *Circovirus* (family *Circoviridae*) comprises, at present, eleven recognized species. Two species, *Porcine circovirus 1* and *Porcine circovirus 2*, occur in mammals, primarily in pigs, while the remaining nine species have been exclusively described in birds so far (2) and include *Beak and feather disease virus* (3), *Canary circovirus* (4), *Duck circovirus* (5, 6), *Finch circovirus* (7), *Goose circovirus* (1), *Gull circovirus* (7), *Pigeon circovirus* (8), *Starling circovirus* (9), and *Swan circovirus* (10). Diseases associated with circovirus infections have mainly been characterized by immunosuppression facilitating opportunistic secondary infections and by feathering disorders (11).

Here, we report the complete genome sequence of circovirus strain 8454V25-1. Viral infection was detected in a zebra finch (*Taeniopygia guttata*) of a flock suffering from increased mortality and opportunistic infections in Germany by amplification and direct sequencing of a fragment of the *rep* gene using a nested broad-spectrum PCR (10). The complete genome sequence was obtained using primers constructed based on conserved regions of finch circovirus (GenBank accession number DQ845075) and canary circovirus (AJ301633) genomes and by primer walking. Sequences were obtained directly from PCR products using dideoxy Sanger technology and were manually assembled based on overlapping regions.

The genome of zebra finch circovirus 8454V25-1 consists of 1,982 nucleotides with a G+C content of 52.0%. Two major ORFs on complementary strands in opposite orientation were identified encoding the viral Rep protein and the Cap protein. An initiation site for rolling-circle amplification with a stem-loop region and a nonanucleotide motif (TAGTATTAC), as well as a tandem repeat of TGGAACC serving as a putative binding site of the viral Rep protein, were detected in the small intergenic region between both ORFs. Phylogenetic analyses based on full-genome comparisons showed that the zebra finch circovirus was most closely related to the finch circovirus found in Gouldian finches (*Chloebia gouldiae*) with a pairwise whole-genome identity of 78.2%. The obtained data will be helpful for further investigations on circovirus infections and immunosuppressive diseases in these popular companion birds.

### Nucleotide sequence accession number.

The genome sequence of zebra finch circovirus 8454V25-1 has been deposited in GenBank under the accession number KP793918.
